# Note upon a Method of Root Filling

**Published:** 1883-10

**Authors:** Charles S. Tomes

**Affiliations:** F.R.S.


### Note Upon a Method of Root Filling.

BY CHARLES S. TOMES, M.A.,F.R.S.

There are certain qualities which a perfect root filling should possess, which cannot be said to be united in any one material in use for the purpose. It should be easy of insertion, for it often has to be introduced into narrow and tortuous channels; at the same time it should be easy of removal, as it is quite impossible to secure uniform success with dead teeth, and intense suffering or the loss of the tooth may be the result of a root filling which cannot be got out in the event of inflammation supervening; gold or tin is neither easy to insert nor remove. It must completely seal the pulp chamber, so that fluids cannot enter from the apical foramen, and this, wool or such things can never do. It should be of bland non-irritating character, as with all care the escape of a little from the apical foramen may occur. For some years I have been in the habit of using occasionally, when I had pretty full confidence in the healthiness of the root, shellac drawn out into fine threads, introduced cold and packed and consolidated with hot nerve canal instruments, but these fillings were very difficult to remove if the occasion arose. It has lately occurred to me that amongst the varieties of solid paraffin, such as is used for imbedding tissue for the purpose of cutting microscopical sections, a perfectly indifferent substance of any required melting point could be found, and that if one which melts at but little above the temperature of that body be selected, it would be easy to introduce it in a fluid condition. If a hypodermic syringe be filled with such a paraffin, to which two or three drops of carbolic acid have been added, it will be found easy to introduce the nozzle of the syringe a little way up the root canal, the syringe having been moderately heated; when the piston is pressed the liquid paraffin will of itself run a good way along the previously dried root. An excess having been left in the pulp chamber and cavity of decay, a heated Donaldson's nerve bristle worked up and down the canal will soon carry the paraffin to the very end of almost any root, and it can be kept fluid in the tooth either by touches of a hot instrument, or blowing at it with a hot air syringe. The root may be filled entirely with paraffin, or filaments of wood, or even threads of cotton wool may be passed up into the melted paraffin; probably the introduction of fine fibres of wood will be found to be an advantage in most cases, or fine wire may be substituted for the wood. So far, I have had every reason to be satisfied with the results. It is much less troublesome than any method of root filling which I had previously practiced, for the first jet sent in by the syringe does so much towards the filling of any but the finest canals; it seals the canals absolutely, so far as experiment out of the mouth can shew; in its introduction the risk of forcing decomposed products out at the end of a root by a piston-action is reduced to a minimum; and it can easily be got out by warm instruments it the need arises.

Whether it will fulfil my expectations time alone will show; there is but little novelty in the idea, but the method of employing some innocuous body of low melting point with a hypodermic syringe may perhaps serve to diminish the tiresome nature of the operation of root filling, so I have communicated this note on the subject.—*The Journal of the British Dental Association.*

